# Insulin Resistance Is Associated with an Unfavorable Serum Lipoprotein Lipid Profile in Women with Newly Diagnosed Gestational Diabetes

**DOI:** 10.3390/biom13030470

**Published:** 2023-03-03

**Authors:** Mikael Huhtala, Tapani Rönnemaa, Kristiina Tertti

**Affiliations:** 1 Department of Obstetrics and Gynecology, University of Turku, FI-20014 Turku, Finland; 2 Department of Obstetrics and Gynecology, Turku University Hospital, Kiinamyllynkatu 4-8, FI-20521 Turku, Finland; 3 Department of Medicine, University of Turku, FI-20014 Turku, Finland; 4 Division of Medicine, Turku University Hospital, Kiinamyllynkatu 4-8, FI-20521 Turku, Finland

**Keywords:** insulin resistance, metabolomics, metabolome, gestational diabetes, GDM

## Abstract

Background: Gestational diabetes (GDM) is associated with various degrees of insulin resistance—a feature related to increased risk of adverse perinatal outcomes. We aimed to determine the previously poorly investigated associations between maternal insulin resistance and serum fasting metabolome at the time of GDM diagnosis. Methods: Serum lipoprotein and amino acid profile was analyzed in 300 subjects with newly diagnosed GDM using a validated nuclear magnetic resonance spectroscopy protocol. Associations between insulin resistance (homeostasis model assessment of insulin resistance, HOMA2-IR) and serum metabolites were examined with linear regression. Results: We found insulin resistance to be associated with a distinct lipid pattern: increased concentration of VLDL triglycerides and phospholipids and total triglycerides. VLDL size was positively related and LDL and HDL sizes were inversely related to insulin resistance. Of fatty acids, increased total fatty acids, relative increase in saturated and monounsaturated fatty acids, and relative decrease in polyunsaturated and omega fatty acids were related to maternal insulin resistance. Conclusions: In newly diagnosed GDM, the association between maternal insulin resistance and serum lipoprotein profile was largely as described in type 2 diabetes. Lifestyle interventions aiming to decrease insulin resistance from early pregnancy could benefit pregnancy outcomes via more advantageous lipid metabolism.

## 1. Introduction

Gestational diabetes (GDM) affects every sixth pregnancy globally [1], and it is associated with adverse perinatal outcomes such as macrosomia, shoulder dystocia, cesarean delivery, preeclampsia, neonatal hypoglycemia, hyperbilirubinemia, and increased need of neonatal intensive care [2].

In early pregnancy, maternal pancreatic beta-cell mass and function are increased already before the physiologic increase in insulin resistance [3]. In cases of inadequate insulin action, to compensate for the increased insulin resistance, maternal hyperglycemia, i.e., GDM, ensues. The origin of hyperglycemia is heterogeneous among GDM patients, with some having especially increased insulin resistance, some having mainly impaired beta-cell function, and some having a mixed pathophysiologic subtype [4]. The risk of perinatal adverse outcomes is related to GDM subtype—many of the GDM-associated complications are more common in insulin-resistant patients [4,5,6]. What factors fundamentally drive these associations between insulin resistance and adverse perinatal outcomes is not well elucidated.

GDM is characterized not only by hyperglycemia, but also by increased serum lipids, amino acids, and ketone bodies [7,8,9,10,11]. Of these metabolites, lipids and amino acids have previously been shown to associate with fetal growth [12,13,14] and duration of gestation [12]. The associations between maternal insulin resistance and metabolome have previously been studied in the HAPO [15,16] and UPBEAT [17] cohorts as well as at least one smaller prospective cohort [18], but the effects of maternal insulin resistance on lipids in different lipoprotein subfractions are not known. To develop more effective and individualized treatments for GDM, the pathophysiology, also regarding hyperlipidemia and amino-acidemia, needs to be more thoroughly understood. As metformin and insulin treatments have partially diverging effects on maternal serum metabolome [12,13], individualizing treatment according to metabolic profile or pathophysiologic subtype (as described by Powe et al. [4]) could possibly yield improved maternal and fetal outcomes.

In this study, we characterized the associations between insulin resistance and maternal serum metabolome in patients with newly diagnosed GDM. We hypothesized that maternal insulin resistance would be associated with similar changes in serum metabolomics as previously seen in type 2 diabetes, and that the changes in serum metabolome in GDM would be largely related to insulin resistance.

## 2. Materials and Methods

The patients in this cohort were derived from a previous randomized trial comparing metformin and insulin treatments of GDM at Turku University Hospital, Finland, during the years 2006–2010 [19]. Patients who met the same inclusion and exclusion criteria but did not require antihyperglycemic medication were also included. The study population was described previously in detail [12,19,20]. Briefly, women with newly diagnosed GDM and singleton pregnancy were included in the study. GDM diagnosis was based on at least 2 abnormal values in a 2 h 75 g oral glucose tolerance test (OGTT). The diagnostic cutoff values were ≥4.8 (fasting), ≥10.0 (1 h), and ≥8.7 mmol/L (2 h) until the release of Finnish national guidelines in December 2008; thereafter cutoff values were ≥5.3, ≥10.0, and ≥8.6 mmol/L, respectively. The exclusion criteria were cardiac or renal insufficiency, liver disease, metformin use 0–3 months prior to conception or during early pregnancy before OGTT, or glucose above 7.0 mmol/L (fasting) or 11.0 mmol/L (60 min postprandial) in home plasma glucose monitoring. Pharmacological treatment (with metformin or insulin [19]) was initiated in cases of recurrent hyperglycemia (fasting glucose ≥ 5.5 and/or postprandial glucose ≥ 7.8 mmol/L) despite diet and lifestyle changes. Patients were recruited at mean 30 gestational weeks, after a diagnostic OGTT. Follow-up and mode and timing of delivery was decided by the managing clinician according to hospital guidelines.

Maternal fasting blood samples were drawn at recruitment after GDM diagnosis and prior to initiation of any pharmacological antihyperglycemic treatment. Fasting plasma glucose, C-peptide, and glycated hemoglobin A1 (HbA1c) were analyzed using routine laboratory methods in the clinical laboratory of Turku University Hospital [19]. Samples for fasting plasma glucose measurement were collected in lithium heparin gel tubes according to local hospital laboratory protocol. All glucose samples were analyzed within 60 min of collection. The homeostasis model assessment of insulin resistance (HOMA2-IR) was calculated using C-peptide and glucose (https://www.dtu.ox.ac.uk/homacalculator, accessed on 3 November 2021) [21]. C-peptide with its longer half-life than insulin is a good measure of insulin secretion and may be used when estimating insulin resistance [22,23].

Additional serum samples were stored at below −70 °C to be used for assessment of targeted maternal metabolome (including detailed lipoprotein profile, fatty acids (FAs), and amino acids) by commercially available high-throughput ^1^H nuclear magnetic resonance spectroscopy (NMR) method (Nightingale Health Ltd., Helsinki, Finland) [24]. After sample preparation (described in [25,26]), a Bruker AVALANCE III 500 Mhz spectrometer was used to acquire the NMR spectra in two parts. First, a presaturated proton NMR spectrum including resonances from proteins and lipids in different lipoprotein particles, and, second, a T2-filtered spectrum in which the broad macromolecule and lipoprotein lipid signals are mostly suppressed, leading to better detection of low-molecular-weight metabolites [24]. The spectra were measured with 80 k data points and 8 scans with Bruker noesypresat pulse sequence, and 64 k data points and 24 or 16 scans with T2-relaxation filtered Bruker 1D CPMG pulse sequence, respectively. The NMR data were processed using Fourier transformation, phase correction, overall signal check for missing and/or extra peaks, background control, removal of baseline, and spectral area-specific signal alignments [26]. As quality control, the results were compared to control samples and an extensive database of quantitative molecular data. The quantification method was based on Bayesian models [27,28], and the concentrations were calibrated to agree with external standards [26]. This method enables quantification of lipid content in different lipoprotein subclasses and has been calibrated against gel permeation high-performance liquid chromatography [29]. The consistency is also similar to mass spectrometry and gas chromatography [30,31].

The original trial was approved by the Ethics Committee of the Southwest Hospital District of Finland (Dnro 246/2005); it is registered at Clinicaltrials.gov (3 November 2010, NCT01240785, http://clinicaltrials.gov/ct2/show/NCT01240785), and it is in accordance with the 1964 Helsinki Declaration. New specimens or clinical data were not collected for the present secondary analysis; therefore, separate ethics committee approval was considered unnecessary. All the study participants signed an informed consent for participating in the original trial and for collection and further analysis of serum samples.

### Statistical Analyses

All analyses were performed in R statistical software (version 4.0.3). Associations between HOMA2-IR and individual serum metabolites were assessed by linear regression. Prior to analyses, HOMA2-IR was log-transformed, centered, and scaled and the metabolite data centered and scaled. Regression models were run unadjusted and adjusted for a priori selected confounding factors: body mass index (BMI) class and gestational age at sampling. BMI classes were normal weight (18.5–24.9 kg/m^2^), overweight (25–30 kg/m^2^), and obese (≥30 kg/m^2^), according to BMI at the first antenatal visit. Two women were underweight (BMI < 18.5 kg/m^2^) and were excluded from the analyses. For illustrative purposes, unadjusted univariate regression analyses were performed also without first centering and scaling the data. Confidence intervals (CI) were acquired with the adjusted bootstrap percentile method. To avoid type I error, a *p*-value below 0.01 was considered statistically significant.

## 3. Results

**Figure 1:**
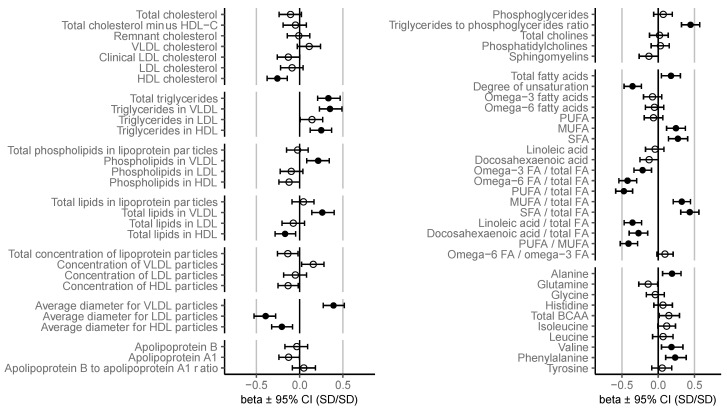
Associations between insulin resistance and serum metabolites. Regression β-estimates with 95% confidence intervals are given for each association adjusted for BMI class and gestational age at sampling. Filled circles denote significant (*p* < 0.01) and open circles nonsignificant associations. BCAA: branched-chain amino acids, FA: fatty acids, MUFA: monounsaturated FAs, PUFA: polyunsaturated FAs, SFA: saturated FAs.

**Figure 2:**
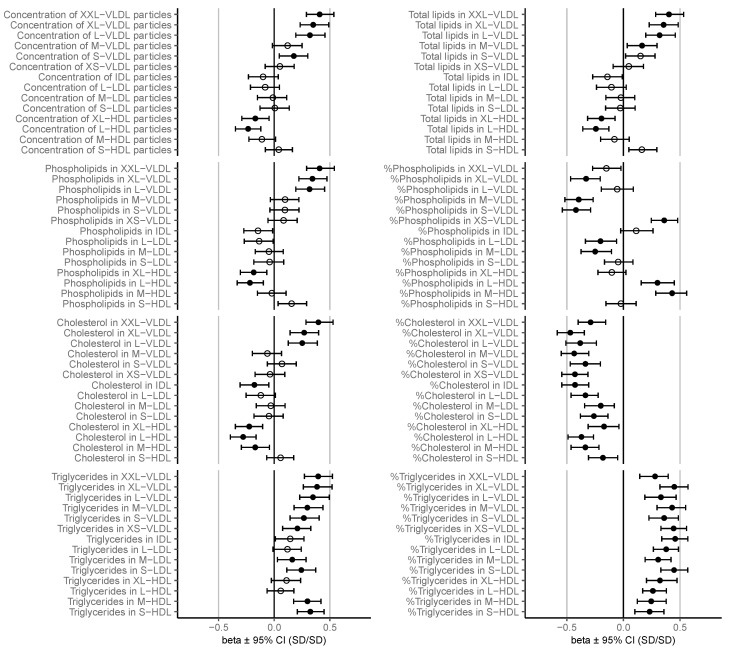
Associations between insulin resistance and lipoprotein lipids. Regression β-estimates with 95% confidence intervals are given for each association adjusted for BMI class and gestational age at sampling. Filled circles denote significant (*p* < 0.01) and open circles nonsignificant associations. S/M/L: small/medium/large, XS: very small, XL: very large, XXL: extremely large.

C-peptide, glucose, and serum samples for NMR analysis were available from 300 patients. Because some individual metabolite measures were discarded in the quality control, and there were missing data regarding BMI, 271–300 patients were available for the unadjusted and 268–297 for the adjusted analyses. Clinical characteristics are given in Table 1. The study participants were, on average, 31.6 ± 5.2 years old with mean pre-pregnancy BMI of 29.2 ± 5.3 kg/m^2^. GDM was diagnosed at mean 26.9 ± 2.4 gestational weeks, and C-peptide and NMR metabolome were measured at mean 30.5 ± 1.8 gestational weeks. Overall, the adjustment for BMI and gestational age had mostly minimal effects on the associations (Table S1). The adjusted associations are depicted in Figure 1 and Figure 2, and the results from both unadjusted and adjusted regression analyses are given in more detail in a supplementary table (Table S1).

### 3.1. Lipoprotein Concentrations and Total Lipids

Insulin resistance was positively related to particle concentration and total lipids in large to extremely large very-low-density lipoprotein (VLDL) (Figure 2), and total VLDL lipids (Figure 1). The positive associations were significant between insulin resistance, total lipids in medium VLDL, and concentration of small VLDL (Figure 2). Insulin resistance was inversely related to particle concentration and concentration of total lipids in large to very large high-density lipoprotein (HDL) (Figure 2).

### 3.2. Cholesterol

While insulin resistance was positively related to the concentrations of cholesterol in large to extremely large VLDL, the association to relative cholesterol content in every lipoprotein subclass was inverse (Figure 2). Insulin resistance was also inversely related to cholesterol concentration in intermediate-density lipoprotein (IDL), total HDL, and medium to very large HDL (Figure 1 and Figure 2).

### 3.3. Triglycerides

Insulin resistance was associated to increased total triglyceride (TG) concentration, increased TG concentration in VLDL and HDL, and increased TG-to-phosphoglycerides ratio (Figure 1). In more detailed lipoprotein subclasses the associations were significant in all VLDL subclasses, small to medium low-density lipoprotein (LDL), and small to medium HDL. Insulin resistance was associated with a notable increase of relative TG content in every lipoprotein subclass (Figure 2).

### 3.4. Phospholipids

Insulin resistance was positively related to the concentration of phospholipids in total VLDL (Figure 1) and large to extremely large VLDL, and inversely to phospholipids in large to very large HDL (Figure 2). The relationship between insulin resistance and relative number of phospholipids in various lipoprotein subclasses contained notable variation regarding magnitude and direction. There was an inverse association between insulin resistance and relative phospholipid content in very large VLDL, medium VLDL, small VLDL, large LDL, and medium LDL and a positive association between insulin resistance and relative number of phospholipids in very small VLDL, large HDL, and medium HDL (Figure 2).

### 3.5. Lipoprotein Particle Size

**Figure 3:**
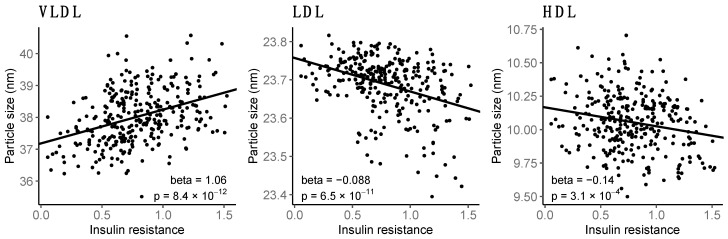
Associations between insulin resistance and average lipoprotein particle size. Insulin resistance, as estimated by log-HOMA2-IR, versus mean particle diameter (nm). Trend line depicts regression beta coefficient with the respective *p*-values.

Insulin resistance related positively to average VLDL particle size and inversely to LDL and HDL particle sizes (Figure 1 and Figure 3).

### 3.6. Fatty Acids (FAs)

Insulin resistance was positively associated with total FAs, saturated FAs (SFAs), and monounsaturated FAs (MUFAs) concentrations (Figure 1). Accordingly, insulin resistance was positively associated with the MUFA-to-total-FA and SFA-to-total-FA ratios, whereas the associations of insulin resistance with the polyunsaturated FA (PUFA)-to-total-FA ratio, PUFA-to-MUFA ratio, and degree of FA unsaturation were inverse. Insulin resistance was inversely related to proportions of linoleic acid, docosahexaenoic acid, omega-3 FAs, and omega-6 FAs (Figure 1).

### 3.7. Amino Acids

Of amino acids, alanine, valine, and phenylalanine were positively related to insulin resistance (Figure 1). Total branched-chain amino acid (BCAA) concentration was positively related to insulin resistance in the unadjusted model, but the association was no longer significant after adjusting for confounding factors (Table S1).

## 4. Discussion

We found insulin resistance in newly diagnosed GDM patients to be associated with increased serum VLDL concentration, total TG concentration, and increased TG content in VLDL, small to medium LDL, and small to medium HDL. Additionally, insulin resistance was related to increased VLDL particle size and decreased HDL and LDL particle sizes. These observations are mostly similar to differences in lipoprotein profile between nonpregnant patients with type 2 diabetes and normoglycemic controls [32]. Additionally, increased concentrations of total FAs, MUFAs, and SFAs and decreased FA unsaturation, PUFA-to-MUFA ratio, and lower proportions of omega-3 FAs, omega-6 FAs, linoleic acid, and docosahexaenoic acid were related to increased insulin resistance.

In the current era of personalized medicine, we are expecting the more detailed classification of GDM to improve outcomes [33], although the exact pathogenesis of GDM remains incompletely understood. The associations between insulin resistance and serum metabolome have been characterized previously outside pregnancy, but to extrapolate these findings into a pregnant population has several pitfalls. In addition, previous studies involving pregnant subjects have been scarce [15,16,17,18], and to our best knowledge the associations between maternal insulin resistance in GDM and detailed lipoprotein lipid profile have not previously been studied.

Previously when stratified according to pathophysiologic subtype of GDM (insulin resistance, beta-cell deficiency, or mixed), total TG was higher and HDL cholesterol lower in the insulin-resistant compared to the beta-cell-deficient subgroup [34]—largely in agreement with our findings. Conversely, the only difference between beta-cell-deficient GDM patients and normoglycemic controls was higher free fatty acid concentration in the former subgroup [34]. Altogether, the literature supports the hypothesis that hypertriglyceridemia in GDM is driven by maternal insulin resistance [15,17,34]. Here we provide further evidence that insulin resistance is related to variation in the same lipoprotein lipids that have previously been reported to differ between GDM patients and normoglycemic pregnant controls, such as higher VLDL particle concentration and lipid contents, increased proportion of TG in most lipoprotein classes, increased cholesterol in largest lipoproteins, and decreased cholesterol large HDL particles [10,11].

**Figure 4:**
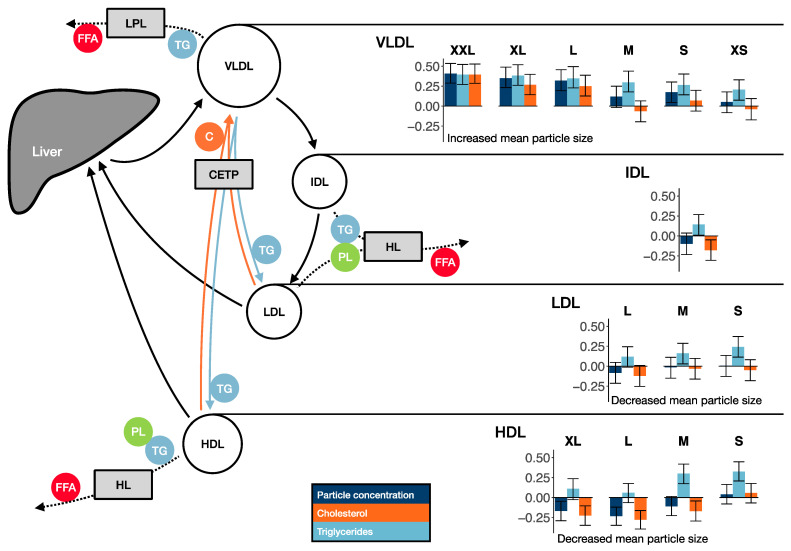
Lipoprotein metabolism and associations between insulin resistance and lipids in lipoprotein subfractions. Very-low-density lipoproteins (VLDLs) are synthesized in the liver. Lipoprotein lipase (LPL) hydrolyzes triglycerides (TGs) in VLDLs to glycerol and free fatty acids (FFAs) to be stored in adipose tissue. VLDLs are transformed into intermediate density lipoproteins (IDLs) and subsequently into low-density lipoproteins (LDLs), which are metabolized in the liver. High-density lipoproteins (HDLs) transport cholesterol (C) from other tissues to liver. Cholesterol ester transfer protein (CETP) exchanges C and TG between LDL, HDL, and VLDL. Hepatic lipase (HL) hydrolyzes TG and phospholipids (PLs) in IDL, LDL, and HDL, hence promoting formation of smaller LDL and HDL particles. The associations between insulin resistance (HOMA2-IR) and particle concentration, C, and TG in different lipoprotein subfractions (XS–XXL) are shown as standardized regression coefficients with 95% confidence intervals. Regression coefficients were adjusted for gestational age at sampling and maternal BMI class.

VLDL, initially rich in TG, are formed in the liver and deliver FAs into the peripheral tissues when VLDL TG is hydrolyzed by lipoprotein lipase (LPL) (Figure 4). VLDL size is decreased by the gradual removal of TG, until VLDL is transformed into IDL. LPL activity is enhanced by insulin and, on the contrary, attenuated by insulin resistance. The observed positive associations between insulin resistance and large VLDL particles likely reflect hepatic insulin resistance and increased VLDL synthesis. Also due to prevailing insulin resistance, the formation of smaller VLDL particles, by LPL, is decreased, and likely hence weaker associations were observed between smaller VLDL lipids and insulin resistance. The positive associations between insulin resistance and VLDL particle size and increased large VLDL concentrations are consistent with the previous literature outside pregnancy [35,36]. The associations to IDL were weaker; only the inverse associations to absolute and relative cholesterol in IDL and positive association to relative amount of TG in IDL were significant. While pregnancy in general is related to increase in total lipids in all VLDL subclasses and IDL and a slight increase in VLDL particle size [37,38], we observed strong positive associations between insulin resistance, lipids in larger VLDL subclasses, and especially VLDL particle size.

Excessive maternal TG-rich VLDL concentrations are likely driven by increased production in the liver and decreased clearance in maternal peripheral tissues due to reduced LPL activity, both related to insulin resistance [39]. However, to which extent maternal insulin resistance affects placental transfer of lipids is currently not known. As lipoproteins cannot simply cross the placenta, TG in lipoproteins is hydrolyzed into FAs, which are then transferred into the placenta [39]. Placental LPL (pLPL) and endothelial lipase are among the most studied lipases in the placenta [40], but due to natural limitations in accessing human placenta, placental lipoprotein metabolism remains incompletely understood. Based on limited evidence, insulin and hyperglycemia may affect pLPL activity [41]. However, given the greater abundance of maternal peripheral LPL and higher activity of LPL in maternal adipose tissue compared to pLPL [42], the effect of placental metabolism on maternal circulating VLDL concentration is assumed to be small.

We found insulin resistance to associate with a higher concentration of cholesterol and phospholipids in large to extremely large VLDL. This observation likely results from higher overall production and decreased clearance of these particles, as the association between insulin resistance and the relative amount of cholesterol and phospholipids in the corresponding particles was inverse or absent. Why insulin resistance was associated with increased proportion of phospholipids in very small VLDL remains to be clarified.

In GDM, increased insulin resistance combined with beta-cell deficiency was associated with higher total cholesterol and LDL cholesterol than sole insulin resistance [34]. We did not find associations between insulin resistance and LDL particle or LDL cholesterol concentrations, but insulin resistance was, however, clearly related to a shift toward smaller LDL particles and increased LDL TG content. This is likely promoted by high VLDL TG, which increases cholesterol ester transfer protein (CETP)-mediated transfer of TG from VLDL to LDL [43]. LDL TG and phospholipids are hydrolyzed by hepatic lipase, leading to a decrease in LDL particle size [44]. Accordingly, we found inverse associations between insulin resistance and relative LDL cholesterol content. LDL particle size is not significantly affected by pregnancy [37], but decreased LDL particle size could be a marker of excessive insulin resistance in pregnancies complicated by GDM as found in the present study.

Generally, pregnancy is related to increased HDL particle size [37,38,45], increased HDL TG [37,46], and altered protein composition of HDL [38]. We found insulin resistance to be related to increased relative TG and decreased relative cholesterol in HDL, but also decreased HDL particle size. Insulin resistance was also associated with increased absolute TG in small to medium HDL and decreased phospholipids, cholesterol, total lipids, and particle concentration in large to very large HDL. In line with our findings, large HDL particles have been inversely associated and small HDL particles positively associated with insulin resistance [35,36]. In GDM, HDL particle size is decreased [10,11]. By which mechanisms HDL particle distribution is altered in obesity, insulin resistance, and diabetes is not fully known.

TG enrichment of HDL has been contributed to increased activity of CETP [44,46], which transfers cholesterol esters from HDL to LDL and VLDL in exchange for TG (Figure 4). Hydrolysis of HDL TG and phospholipids by hepatic lipase promotes formation of smaller HDL particles [32,44]. While increase in HDL TG is physiologic in pregnancy, TG in small and medium HDL are further increased in obese women with GDM compared to normoglycemic controls [10,11].

Insulin resistance in our study was not related to either apolipoprotein A1 (ApoA1), the major apolipoprotein in HDL particles, or apolipoprotein B (ApoB), the major apolipoprotein in TG-rich lipoprotein VLDL and LDL. One copy of ApoB-100 is found in each VLDL, IDL, and LDL particle. Given that LDL is the most abundant of these, the ApoB measure very closely reflects the concentration of LDL. Accordingly, the associations between insulin resistance, ApoB, and ApoA1 resembled the associations between insulin resistance, LDL particle concentration, and HDL particle concentration, respectively. None of those associations were statistically significant in our study. Outside pregnancy, insulin resistance has been related to LDL particle concentrations [35,47], and in a cross-sectional design the apoB-to-ApoA1 ratio has been associated with insulin resistance [48]. Altogether, our data underscore the relationship between insulin resistance and the particle size and TG content of HDL and LDL, rather than mere particle concentrations.

There was a clear association between insulin resistance and FA classes. SFAs and MUFAs were positively related and PUFAs, omega-3 FAs, and omega-6 FAs inversely related to insulin resistance. Similarly, in the first trimester, maternal insulin resistance is associated with lower omega-3 FAs [49]. Although all analyses in the present study were made assuming insulin resistance to be the predictor, the causality might be to some extent vice versa. Circulating free FAs have been shown to promote insulin resistance [50,51]. Based on our data, the overall FA profile and insulin resistance are highly intercorrelated in GDM. However, supplementation with fish oil seems not to improve insulin resistance [52].

In women with GDM, the ratios of omega-6 FAs, linoleic acid, and PUFAs to total FAs were significantly lower and the MUFA-to-total-FA ratio higher compared to normoglycemic pregnant controls [10,11]. The differences in absolute concentrations of the FA classes and in the omega-3-to-total-FA ratio were clearly smaller [10,11]. These associations are similar to our observations in associations between insulin resistance and FA classes. During late pregnancy, lipolysis increases so that the circulating FAs may represent mobilization of maternal FA stores. Additionally, in late pregnancy fetal FAs are obtained increasingly via de novo lipogenesis and less by placental transfer [53], although the essential FAs need to be obtained from the mother. We have previously demonstrated that maternal total FAs, SFAs, and MUFAs are related to higher birth weight [13], but whether the proportion of certain FA classes in maternal circulation are of importance regarding fetal growth or perinatal outcomes requires further studies.

Although BCAAs are strongly associated to insulin resistance in general [54] and in pregnancy [15], we found insulin resistance to be related positively to total BCAAs only in the unadjusted model. After accounting for confounding factors, valine was the only BCAA associated with insulin resistance. These differences between our data and previous literature [15] might be explained by the smaller sample size in our study, or that all patients in our study had GDM and most required antihyperglycemic medication. Additionally, blood samples were not collected on a fixed gestational age in our study, which may have produced some degree of variation in the data. However, we found clear positive associations between insulin resistance and alanine and phenylalanine, comparable to what was previously reported [15].

Altogether, these findings in recently diagnosed GDM patients demonstrate that the associations between insulin resistance and serum metabolites are similar in GDM compared to those in nonpregnant subjects with type 2 diabetes [32]. Moreover, insulin resistance could significantly contribute to differences in lipoprotein lipid profile observed between GDM patients and pregnant normoglycemic controls [10,11]. Lifestyle interventions have been capable of ameliorating insulin resistance [55], and in one study such an intervention has even reduced the risk of developing GDM, when deployed early in pregnancy [56]. Therefore, focusing on interventions in early pregnancy, or even before conception, to decrease insulin resistance in high-risk populations could yield not only decreased risk of GDM, but also overall healthier metabolic profile with plausible additional benefits. 

### Strengths and Limitations

The strengths of our study are reasonably large sample size, prospectively collected data, and the use of a validated and widely used NMR protocol. Still, this was a secondary analysis of previously collected data, and drawing all serum samples at the same gestational age could have reduced some variation in the data. Moreover, other metabolites such as acylcarnitines and all individual FAs were not included in the targeted NMR metabolome. We estimated insulin resistance using HOMA2-IR, which is not as precise as hyperinsulinemic clamp. HOMA estimates of insulin resistance are, however, frequently used in research [4,15,16,18] due to the method being less invasive, less time-consuming, and more applicable for studies with a higher number of participants. Use of lithium heparin gel tubes, compared to fluoride citrate tubes [57], for fasting glucose measurement may have resulted in slightly lower glucose values in our analyses. It is unlikely that this has caused any systematic bias in our regression analyses, as the HOMA2-IR values were centered and scaled prior to analyses.

## 5. Conclusions

As expected, we found strong positive associations between insulin resistance and serum TG rich lipoproteins in GDM. Moreover, the FA profile was related to insulin resistance. The associations between insulin resistance and fasting amino acids were weaker than expected. These associations were similar to those previously described in type 2 diabetes, but we demonstrated their relevance also in the pregnant population with GDM. Furthermore, small LDL particle size could be a marker of strong insulin resistance in GDM pregnancy. Lifestyle interventions aiming to lower insulin resistance from early pregnancy or before conception could benefit pregnancy outcomes via more advantageous lipid metabolism.

## Figures and Tables

**Table 1:** Population characteristics.

	Mean ± SD or n (%)	Range	n
Age, years	31.6 ± 5.2	19.0; 44.0	300
Pre-pregnancy BMI, kg/m^2^	29.2 ± 5.3	19.0; 52.0	297
Nulliparous, n (%)	128 (42.7)		300
Smoking, n (%)	32 (10.9)		294
Gestational age at OGTT, weeks	26.9 ± 2.4	12.6; 31.6	299
OGTT fasting glucose, mmol/L	5.5 ± 0.5	4.0; 7.1	300
OGTT 1 h glucose, mmol/L	11.1 ± 1.3	4.6; 14.9	300
OGTT 2 h glucose, mmol/L	8.0 ± 1.8	4.3; 16.3	298
HbA1c, %	5.5 ± 0.3	4.6; 6.3	300
HbA1c, mmol/mol	36.1 ± 3.6	26.8; 45.3	300
Gestational age at sampling, weeks	30.5 ± 1.8	22.1; 34.6	300
C-peptide, nmol/L	1.04 ± 0.31	0.50; 2.00	300
HOMA2-IR	2.3 ± 0.7	1.1; 4.6	300

BMI: body mass index, OGTT: oral glucose tolerance test, HbA1c: glycated hemoglobin A1, HOMA2-IR: homeostasis model assessment 2—insulin resistance.

## Data Availability

The data that support the findings of this study are available from the corresponding author, M.H., upon reasonable request.

## Supplementary Materials

The following supporting information can be downloaded at: https://www.mdpi.com/article/10.3390/biom13030470/s1; Supplementary Table S1–Unadjusted and adjusted associations between insulin resistance and serum metabolites, and mean concentrations of serum metabolites.
