# Supplementary material for: Insulin Resistance Is Associated with an Unfavorable Serum Lipoprotein Lipid Profile in Women with Newly Diagnosed Gestational Diabetes
Source: Biomolecules. 2023 Mar 3;13(3):470. doi: 10.3390/biom13030470 (PMC10046655; doi:10.3390/biom13030470)
Supplement: Supplementary file 1 [file biomolecules-13-00470-s001.zip › biomolecules-2239936-supplementary.pdf]

Supplementary table S1 – Unadjusted and adjusted associations between insulin resistance and serum metabolites, and mean concentrations of serum metabolites

Regression coefficients values (beta) with 95% confidence intervals (CI) are given for unadjusted model and model adjusted for BMI class and gestational age at sampling. VLDL: very-low-density lipoprotein, LDL: low-density lipoprotein, IDL: intermediate-density lipoprotein, HDL: high-density lipoprotein. FA: fatty acids, SFA: saturated FA, MUFA: monounsaturated FA, PUFA: polyunsaturated FA, BCAA: branched-chain amino acids, S/M/L: small/medium/large, XS: very small, XL: very large, XXL: extremely large.

				Unadjusted model			Adjusted model		
**Metabolite**	**Mean ± SD**	**unit**	**n**	**beta [95% CI]**	**p-value**	**n**	**beta [95% CI]**	**p-value**	**n**
Total cholesterol	6.61 ± 1.29	mmol/l	300	-0.098 [-0.22; 0.023]	0.091	300	-0.11 [-0.24; 0.019]	0.094	297
Total cholesterol minus HDL-C	5.18 ± 1.29	mmol/l	300	-0.046 [-0.17; 0.072]	0.43	300	-0.049 [-0.19; 0.078]	0.44	297
Remnant cholesterol	2.69 ± 0.67	mmol/l	300	-0.012 [-0.13; 0.11]	0.84	300	-0.010 [-0.14; 0.12]	0.87	297
VLDL cholesterol	1.45 ± 0.42	mmol/l	300	0.10 [-0.019; 0.22]	0.082	300	0.11 [-0.028; 0.24]	0.080	297
Clinical LDL cholesterol	3.73 ± 1.08	mmol/l	300	-0.12 [-0.24; -0.000]	0.035	300	-0.13 [-0.26; 0.001]	0.038	297
LDL cholesterol	2.49 ± 0.63	mmol/l	300	-0.082 [-0.20; 0.042]	0.16	300	-0.089 [-0.22; 0.039]	0.16	297
HDL cholesterol	1.43 ± 0.28	mmol/l	300	-0.23 [-0.34; -0.13]	<0.0001	300	-0.26 [-0.38; -0.14]	<0.0001	297
Total triglycerides	2.27 ± 0.75	mmol/l	300	0.31 [0.20; 0.42]	<0.0001	300	0.33 [0.21; 0.47]	<0.0001	297
Triglycerides in VLDL	1.50 ± 0.63	mmol/l	300	0.33 [0.23; 0.44]	<0.0001	300	0.35 [0.23; 0.49]	<0.0001	297
Triglycerides in LDL	301 ± 68	µmol/l	300	0.13 [0.011; 0.24]	0.022	300	0.14 [0.005; 0.27]	0.023	297
Triglycerides in HDL	250 ± 52	µmol/l	300	0.23 [0.12; 0.34]	<0.0001	300	0.25 [0.12; 0.37]	<0.0001	297
Total phospholipids in lipoprotein particles	3.80 ± 0.52	mmol/l	300	-0.017 [-0.14; 0.099]	0.77	300	-0.024 [-0.15; 0.099]	0.71	297
Phospholipids in VLDL	855 ± 256	µmol/l	300	0.20 [0.081; 0.32]	0.00050	300	0.21 [0.080; 0.34]	0.00061	297
Phospholipids in LDL	795 ± 182	µmol/l	300	-0.090 [-0.20; 0.029]	0.12	300	-0.097 [-0.23; 0.038]	0.12	297
Phospholipids in HDL	1.70 ± 0.29	mmol/l	300	-0.11 [-0.22; 0.005]	0.065	300	-0.12 [-0.24; -0.000]	0.056	297
Total lipids in lipoprotein particles	12.7 ± 2.2	mmol/l	300	0.045 [-0.077; 0.16]	0.44	300	0.043 [-0.090; 0.16]	0.49	297
Total lipids in VLDL	3.81 ± 1.23	mmol/l	300	0.25 [0.13; 0.36]	<0.0001	300	0.26 [0.14; 0.40]	<0.0001	297
Total lipids in LDL	3.58 ± 0.86	mmol/l	300	-0.068 [-0.19; 0.048]	0.24	300	-0.074 [-0.20; 0.057]	0.24	297
Total lipids in HDL	3.39 ± 0.57	mmol/l	300	-0.15 [-0.26; -0.041]	0.0090	300	-0.17 [-0.29; -0.047]	0.0081	297
Total concentration of lipoprotein particles	23.8 ± 2.8	µmol/l	300	-0.11 [-0.22; -0.004]	0.052	300	-0.14 [-0.26; -0.020]	0.032	297
Concentration of VLDL particles	387 ± 105	nmol/l	300	0.14 [0.026; 0.26]	0.013	300	0.16 [0.022; 0.28]	0.012	297
Concentration of LDL particles	1.84 ± 0.49	µmol/l	300	-0.046 [-0.17; 0.071]	0.42	300	-0.051 [-0.19; 0.079]	0.42	297
Concentration of HDL particles	21.0 ± 2.7	µmol/l	300	-0.11 [-0.22; -0.005]	0.059	300	-0.13 [-0.25; -0.017]	0.035	297
Average diameter for VLDL particles	38.0 ± 0.9	nm	300	0.38 [0.28; 0.49]	<0.0001	300	0.39 [0.27; 0.52]	<0.0001	297
Average diameter for LDL particles	23.7 ± 0.1	nm	300	-0.37 [-0.49; -0.26]	<0.0001	300	-0.39 [-0.53; -0.28]	<0.0001	297
Average diameter for HDL particles	10.1 ± 0.2	nm	300	-0.21 [-0.32; -0.098]	0.00031	300	-0.21 [-0.33; -0.083]	0.0012	297
Phosphoglycerides	3.39 ± 0.51	mmol/l	298	0.070 [-0.055; 0.19]	0.23	298	0.069 [-0.063; 0.19]	0.28	295
Triglycerides to phosphoglycerides ratio	0.66 ± 0.16	ratio	298	0.42 [0.32; 0.53]	<0.0001	298	0.45 [0.32; 0.57]	<0.0001	295
Total cholines	3.66 ± 0.54	mmol/l	299	0.025 [-0.10; 0.14]	0.67	299	0.018 [-0.12; 0.14]	0.77	296
Phosphatidylcholines	3.25 ± 0.49	mmol/l	299	0.033 [-0.089; 0.15]	0.58	299	0.030 [-0.098; 0.15]	0.64	296
Sphingomyelins	635 ± 105	µmol/l	299	-0.10 [-0.23; 0.015]	0.076	299	-0.13 [-0.26; -0.001]	0.045	296
Apolipoprotein B	1.44 ± 0.38	g/l	300	-0.032 [-0.16; 0.086]	0.58	300	-0.033 [-0.17; 0.092]	0.60	297
Apolipoprotein A1	1.51 ± 0.25	g/l	300	-0.11 [-0.22; -0.004]	0.052	300	-0.13 [-0.24; -0.004]	0.045	297
Apolipoprotein B to apolipoprotein A1 ratio	0.98 ± 0.33	ratio	300	0.040 [-0.080; 0.16]	0.49	300	0.047 [-0.085; 0.18]	0.46	297
Total fatty acids	17.5 ± 2.7	mmol/l	299	0.17 [0.051; 0.29]	0.0029	299	0.18 [0.043; 0.31]	0.0053	296
Degree of unsaturation	1.33 ± 0.09	degree	299	-0.32 [-0.43; -0.20]	<0.0001	299	-0.35 [-0.47; -0.23]	<0.0001	296
Omega-3 fatty acids	785 ± 226	µmol/l	299	-0.067 [-0.19; 0.047]	0.25	299	-0.079 [-0.20; 0.049]	0.22	296
Omega-6 fatty acids	5.63 ± 0.69	mmol/l	299	-0.043 [-0.16; 0.074]	0.46	299	-0.052 [-0.18; 0.076]	0.42	296
PUFA	6.41 ± 0.82	mmol/l	299	-0.054 [-0.18; 0.064]	0.35	299	-0.065 [-0.19; 0.064]	0.31	296
MUFA	4.89 ± 0.99	mmol/l	299	0.25 [0.13; 0.37]	<0.0001	299	0.24 [0.12; 0.37]	<0.0001	296
SFA	6.15 ± 1.02	mmol/l	299	0.25 [0.13; 0.37]	<0.0001	299	0.27 [0.14; 0.41]	<0.0001	296
Linoleic acid	4.95 ± 0.79	mmol/l	299	-0.040 [-0.16; 0.078]	0.49	299	-0.044 [-0.18; 0.079]	0.50	296
Docosahexaenoic acid	441 ± 94	µmol/l	299	-0.11 [-0.23; 0.015]	0.065	299	-0.13 [-0.25; 0.002]	0.051	296
Omega-3 FA / total FA	4.49 ± 1.10	%	299	-0.20 [-0.32; -0.094]	0.00038	299	-0.22 [-0.34; -0.094]	0.00070	296
Omega-6 FA / total FA	32.5 ± 2.5	%	299	-0.41 [-0.51; -0.30]	<0.0001	299	-0.42 [-0.54; -0.30]	<0.0001	296
PUFA / total FA	37.0 ± 2.8	%	299	-0.45 [-0.56; -0.35]	<0.0001	299	-0.47 [-0.59; -0.35]	<0.0001	296
MUFA / total FA	27.8 ± 2.1	%	299	0.35 [0.25; 0.47]	<0.0001	299	0.33 [0.21; 0.44]	<0.0001	296

SFA / total FA	35.2 ± 1.4	%	299	0.36 [0.25; 0.46]	<0.0001	299	0.43 [0.31; 0.56]	<0.0001	296
Linoleic acid / total FA	28.4 ± 2.6	%	299	-0.35 [-0.45; -0.24]	<0.0001	299	-0.35 [-0.47; -0.23]	<0.0001	296
Docosahexaenoic acid / total FA	2.55 ± 0.53	%	299	-0.25 [-0.38; -0.14]	<0.0001	299	-0.27 [-0.40; -0.15]	<0.0001	296
PUFA / MUFA	1.34 ± 0.20	ratio	299	-0.42 [-0.53; -0.31]	<0.0001	299	-0.41 [-0.52; -0.28]	<0.0001	296
Omega-6 FA / omega-3 FA	7.71 ± 2.20	ratio	300	0.088 [-0.021; 0.20]	0.13	300	0.093 [-0.017; 0.21]	0.15	297
Alanine	362 ± 54	μmol/l	300	0.12 [-0.002; 0.25]	0.032	300	0.19 [0.060; 0.31]	0.0027	297
Glutamine	425 ± 63	μmol/l	298	-0.11 [-0.23; 0.007]	0.052	298	-0.14 [-0.27; -0.010]	0.032	295
Glycine	188 ± 30	μmol/l	300	-0.044 [-0.16; 0.077]	0.45	300	-0.040 [-0.16; 0.083]	0.53	297
Histidine	83.4 ± 9.9	μmol/l	300	-0.011 [-0.12; 0.11]	0.85	300	0.066 [-0.061; 0.20]	0.30	297
Total BCAA	314 ± 43	μmol/l	300	0.15 [0.029; 0.28]	0.0089	300	0.15 [0.013; 0.29]	0.021	297
Isoleucine	48.0 ± 9.5	μmol/l	300	0.13 [0.021; 0.24]	0.028	300	0.12 [-0.006; 0.24]	0.069	297
Leucine	93.7 ± 15.2	μmol/l	300	0.099 [-0.021; 0.23]	0.087	300	0.067 [-0.077; 0.20]	0.30	297
Valine	173 ± 23	μmol/l	300	0.16 [0.041; 0.30]	0.0045	300	0.18 [0.044; 0.34]	0.0041	297
Phenylalanine	81.9 ± 13.8	μmol/l	300	0.28 [0.16; 0.40]	<0.0001	300	0.23 [0.10; 0.39]	0.00017	297
Tyrosine	46.2 ± 6.1	μmol/l	300	0.076 [-0.046; 0.20]	0.19	300	0.055 [-0.089; 0.19]	0.39	297
Glycoprotein acetyls	1.01 ± 0.09	mmol/l	300	0.43 [0.33; 0.54]	<0.0001	300	0.40 [0.29; 0.51]	<0.0001	297
Concentration of XXL-VLDL particles	3.11 ± 2.22	nmol/l	300	0.39 [0.29; 0.50]	<0.0001	300	0.41 [0.29; 0.54]	<0.0001	297
Total lipids in XXL-VLDL	279 ± 216	μmol/l	300	0.39 [0.29; 0.50]	<0.0001	300	0.40 [0.28; 0.53]	<0.0001	297
Phospholipids in XXL-VLDL	39.6 ± 28.5	μmol/l	300	0.39 [0.29; 0.50]	<0.0001	300	0.41 [0.29; 0.54]	<0.0001	297
Cholesterol in XXL-VLDL	83.4 ± 51.2	μmol/l	300	0.38 [0.27; 0.48]	<0.0001	300	0.40 [0.29; 0.53]	<0.0001	297
Triglycerides in XXL-VLDL	156 ± 139	μmol/l	300	0.39 [0.29; 0.50]	<0.0001	300	0.39 [0.27; 0.52]	<0.0001	297
Concentration of XL-VLDL particles	9.24 ± 4.23	nmol/l	300	0.33 [0.23; 0.45]	<0.0001	300	0.35 [0.23; 0.49]	<0.0001	297
Total lipids in XL-VLDL	359 ± 175	μmol/l	300	0.34 [0.23; 0.45]	<0.0001	300	0.36 [0.23; 0.48]	<0.0001	297
Phospholipids in XL-VLDL	74.5 ± 33.5	μmol/l	300	0.32 [0.22; 0.44]	<0.0001	300	0.34 [0.22; 0.47]	<0.0001	297
Cholesterol in XL-VLDL	109 ± 44	μmol/l	300	0.25 [0.14; 0.36]	<0.0001	300	0.27 [0.14; 0.40]	<0.0001	297
Triglycerides in XL-VLDL	176 ± 101	μmol/l	300	0.37 [0.26; 0.48]	<0.0001	300	0.38 [0.26; 0.52]	<0.0001	297
Concentration of L-VLDL particles	25.8 ± 10.9	nmol/l	300	0.30 [0.19; 0.42]	<0.0001	300	0.32 [0.19; 0.46]	<0.0001	297
Total lipids in L-VLDL	595 ± 257	μmol/l	300	0.30 [0.19; 0.42]	<0.0001	300	0.32 [0.20; 0.46]	<0.0001	297
Phospholipids in L-VLDL	122 ± 52	μmol/l	300	0.30 [0.19; 0.41]	<0.0001	300	0.32 [0.19; 0.45]	<0.0001	297
Cholesterol in L-VLDL	199 ± 78	μmol/l	300	0.23 [0.12; 0.35]	<0.0001	300	0.25 [0.13; 0.39]	<0.0001	297
Triglycerides in L-VLDL	274 ± 132	μmol/l	300	0.33 [0.22; 0.45]	<0.0001	300	0.35 [0.23; 0.50]	<0.0001	297
Concentration of M-VLDL particles	77.1 ± 24.3	nmol/l	300	0.11 [-0.006; 0.23]	0.049	300	0.12 [-0.017; 0.25]	0.056	297
Total lipids in M-VLDL	960 ± 303	μmol/l	300	0.15 [0.043; 0.27]	0.0073	300	0.16 [0.033; 0.30]	0.0086	297
Phospholipids in M-VLDL	206 ± 64	μmol/l	300	0.093 [-0.028; 0.21]	0.11	300	0.098 [-0.035; 0.22]	0.12	297
Cholesterol in M-VLDL	295 ± 103	μmol/l	300	-0.060 [-0.18; 0.064]	0.30	300	-0.061 [-0.20; 0.066]	0.33	297
Triglycerides in M-VLDL	459 ± 169	μmol/l	300	0.28 [0.17; 0.40]	<0.0001	300	0.30 [0.18; 0.44]	<0.0001	297
Concentration of S-VLDL particles	110 ± 32	nmol/l	300	0.16 [0.050; 0.28]	0.0054	300	0.18 [0.046; 0.30]	0.0047	297
Total lipids in S-VLDL	811 ± 228	μmol/l	300	0.14 [0.021; 0.25]	0.018	300	0.15 [0.019; 0.28]	0.016	297
Phospholipids in S-VLDL	175 ± 50	μmol/l	300	0.087 [-0.035; 0.20]	0.13	300	0.096 [-0.039; 0.22]	0.12	297
Cholesterol in S-VLDL	348 ± 103	μmol/l	300	0.059 [-0.063; 0.18]	0.31	300	0.069 [-0.061; 0.20]	0.27	297
Triglycerides in S-VLDL	287 ± 84	μmol/l	300	0.24 [0.13; 0.37]	<0.0001	300	0.27 [0.14; 0.40]	<0.0001	297
Concentration of XS-VLDL particles	161 ± 37	nmol/l	300	0.041 [-0.078; 0.15]	0.47	300	0.051 [-0.081; 0.18]	0.42	297
Total lipids in XS-VLDL	806 ± 175	μmol/l	300	0.037 [-0.084; 0.16]	0.52	300	0.047 [-0.091; 0.18]	0.45	297
Phospholipids in XS-VLDL	237 ± 53	μmol/l	300	0.071 [-0.050; 0.19]	0.22	300	0.084 [-0.056; 0.21]	0.18	297
Cholesterol in XS-VLDL	418 ± 92	μmol/l	300	-0.044 [-0.16; 0.076]	0.45	300	-0.038 [-0.17; 0.095]	0.54	297
Triglycerides in XS-VLDL	151 ± 36	μmol/l	300	0.19 [0.071; 0.30]	0.00098	300	0.21 [0.075; 0.33]	0.00079	297
Concentration of IDL particles	571 ± 157	nmol/l	300	-0.100 [-0.22; 0.025]	0.085	300	-0.10 [-0.23; 0.036]	0.11	297
Total lipids in IDL	1.90 ± 0.42	mmol/l	300	-0.14 [-0.26; -0.019]	0.019	300	-0.14 [-0.27; -0.010]	0.025	297
Phospholipids in IDL	446 ± 91	μmol/l	300	-0.14 [-0.26; -0.022]	0.015	300	-0.15 [-0.27; -0.014]	0.021	297
Cholesterol in IDL	1.24 ± 0.30	mmol/l	300	-0.17 [-0.29; -0.052]	0.0031	300	-0.18 [-0.31; -0.049]	0.0048	297
Triglycerides in IDL	213 ± 46	μmol/l	300	0.13 [0.006; 0.24]	0.024	300	0.14 [0.010; 0.27]	0.022	297
Concentration of L-LDL particles	1.05 ± 0.29	μmol/l	300	-0.075 [-0.20; 0.039]	0.19	300	-0.082 [-0.21; 0.047]	0.20	297
Total lipids in L-LDL	2.23 ± 0.53	mmol/l	300	-0.098 [-0.22; 0.019]	0.089	300	-0.11 [-0.24; 0.024]	0.094	297
Phospholipids in L-LDL	452 ± 104	μmol/l	300	-0.13 [-0.24; -0.005]	0.029	300	-0.14 [-0.27; -0.009]	0.033	297
Cholesterol in L-LDL	1.57 ± 0.39	mmol/l	300	-0.11 [-0.23; 0.010]	0.052	300	-0.12 [-0.25; 0.010]	0.056	297
Triglycerides in L-LDL	206 ± 46	μmol/l	300	0.11 [-0.005; 0.22]	0.058	300	0.12 [-0.012; 0.24]	0.058	297

Concentration of M-LDL particles	476 ± 129	nmol/l	300	-0.009 [-0.13; 0.10]	0.87	300	-0.012 [-0.15; 0.11]	0.85	297
Total lipids in M-LDL	907 ± 240	µmol/l	300	-0.019 [-0.14; 0.11]	0.75	300	-0.023 [-0.16; 0.10]	0.72	297
Phospholipids in M-LDL	210 ± 53	µmol/l	300	-0.043 [-0.16; 0.073]	0.46	300	-0.048 [-0.17; 0.081]	0.45	297
Cholesterol in M-LDL	629 ± 174	µmol/l	300	-0.027 [-0.15; 0.090]	0.65	300	-0.031 [-0.16; 0.098]	0.62	297
Triglycerides in M-LDL	67.9 ± 15.9	µmol/l	300	0.15 [0.031; 0.26]	0.0084	300	0.16 [0.029; 0.29]	0.0095	297
Concentration of S-LDL particles	313 ± 73	nmol/l	300	0.006 [-0.11; 0.13]	0.92	300	0.005 [-0.13; 0.14]	0.93	297
Total lipids in S-LDL	443 ± 101	µmol/l	300	-0.025 [-0.14; 0.094]	0.67	300	-0.028 [-0.16; 0.10]	0.66	297
Phospholipids in S-LDL	132 ± 27	µmol/l	300	-0.041 [-0.16; 0.075]	0.48	300	-0.041 [-0.18; 0.085]	0.51	297
Cholesterol in S-LDL	284 ± 69	µmol/l	300	-0.043 [-0.16; 0.073]	0.46	300	-0.047 [-0.18; 0.081]	0.45	297
Triglycerides in S-LDL	26.9 ± 6.7	µmol/l	300	0.23 [0.12; 0.35]	<0.0001	300	0.24 [0.11; 0.37]	<0.0001	297
Concentration of XL-HDL particles	763 ± 197	nmol/l	300	-0.17 [-0.28; -0.058]	0.0030	300	-0.17 [-0.29; -0.046]	0.0077	297
Total lipids in XL-HDL	379 ± 105	µmol/l	300	-0.20 [-0.30; -0.084]	0.00068	300	-0.19 [-0.32; -0.074]	0.0023	297
Phospholipids in XL-HDL	193 ± 59	µmol/l	300	-0.19 [-0.30; -0.079]	0.0012	300	-0.18 [-0.30; -0.066]	0.0037	297
Cholesterol in XL-HDL	169 ± 44	µmol/l	300	-0.22 [-0.34; -0.12]	<0.0001	300	-0.23 [-0.35; -0.10]	0.00038	297
Triglycerides in XL-HDL	16.9 ± 4.0	µmol/l	300	0.096 [-0.019; 0.21]	0.098	300	0.11 [-0.026; 0.24]	0.081	297
Concentration of L-HDL particles	3.68 ± 1.17	µmol/l	300	-0.22 [-0.33; -0.12]	<0.0001	300	-0.23 [-0.35; -0.12]	0.00022	297
Total lipids in L-HDL	1.05 ± 0.33	mmol/l	300	-0.23 [-0.34; -0.13]	<0.0001	300	-0.24 [-0.36; -0.13]	0.00012	297
Phospholipids in L-HDL	497 ± 154	µmol/l	300	-0.21 [-0.32; -0.11]	0.00029	300	-0.22 [-0.33; -0.098]	0.00057	297
Cholesterol in L-HDL	488 ± 172	µmol/l	300	-0.26 [-0.38; -0.17]	<0.0001	300	-0.28 [-0.39; -0.16]	<0.0001	297
Triglycerides in L-HDL	66.6 ± 14.3	µmol/l	300	0.046 [-0.065; 0.16]	0.43	300	0.058 [-0.064; 0.18]	0.36	297
Concentration of M-HDL particles	5.42 ± 1.07	µmol/l	300	-0.087 [-0.20; 0.019]	0.13	300	-0.11 [-0.23; 0.013]	0.083	297
Total lipids in M-HDL	944 ± 185	µmol/l	300	-0.055 [-0.17; 0.058]	0.34	300	-0.079 [-0.20; 0.052]	0.21	297
Phospholipids in M-HDL	442 ± 83	µmol/l	300	-0.001 [-0.10; 0.12]	0.98	300	-0.022 [-0.15; 0.11]	0.73	297
Cholesterol in M-HDL	416 ± 106	µmol/l	300	-0.14 [-0.25; -0.031]	0.014	300	-0.17 [-0.29; -0.043]	0.0069	297
Triglycerides in M-HDL	86.3 ± 18.2	µmol/l	300	0.28 [0.16; 0.38]	<0.0001	300	0.30 [0.17; 0.42]	<0.0001	297
Concentration of S-HDL particles	11.2 ± 1.5	µmol/l	300	0.063 [-0.050; 0.17]	0.28	300	0.041 [-0.080; 0.16]	0.52	297
Total lipids in S-HDL	1.01 ± 0.12	mmol/l	300	0.18 [0.073; 0.29]	0.0018	300	0.16 [0.048; 0.30]	0.010	297
Phospholipids in S-HDL	572 ± 72	µmol/l	300	0.17 [0.065; 0.29]	0.0026	300	0.16 [0.035; 0.29]	0.014	297
Cholesterol in S-HDL	360 ± 54	µmol/l	300	0.078 [-0.034; 0.19]	0.18	300	0.057 [-0.067; 0.18]	0.37	297
Triglycerides in S-HDL	80.2 ± 18.9	µmol/l	300	0.30 [0.19; 0.41]	<0.0001	300	0.32 [0.21; 0.45]	<0.0001	297
%Phospholipids in XXL-VLDL	14.9 ± 2.0	%	271	-0.19 [-0.30; -0.081]	0.0019	271	-0.15 [-0.27; -0.022]	0.023	268
%Cholesterol in XXL-VLDL	35.1 ± 11.2	%	271	-0.31 [-0.41; -0.20]	<0.0001	271	-0.29 [-0.40; -0.16]	<0.0001	268
%Triglycerides in XXL-VLDL	50.0 ± 12.7	%	271	0.30 [0.19; 0.40]	<0.0001	271	0.28 [0.14; 0.40]	<0.0001	268
%Phospholipids in XL-VLDL	21.2 ± 1.7	%	299	-0.34 [-0.47; -0.23]	<0.0001	299	-0.33 [-0.47; -0.20]	<0.0001	296
%Cholesterol in XL-VLDL	31.8 ± 5.2	%	299	-0.47 [-0.58; -0.36]	<0.0001	299	-0.47 [-0.58; -0.35]	<0.0001	296
%Triglycerides in XL-VLDL	47.0 ± 6.7	%	299	0.45 [0.34; 0.57]	<0.0001	299	0.45 [0.32; 0.57]	<0.0001	296
%Phospholipids in L-VLDL	20.6 ± 1.0	%	300	-0.053 [-0.18; 0.074]	0.36	300	-0.055 [-0.19; 0.088]	0.39	297
%Cholesterol in L-VLDL	34.2 ± 3.9	%	300	-0.38 [-0.49; -0.26]	<0.0001	300	-0.38 [-0.51; -0.24]	<0.0001	297
%Triglycerides in L-VLDL	45.3 ± 4.7	%	300	0.33 [0.20; 0.44]	<0.0001	300	0.33 [0.19; 0.47]	<0.0001	297
%Phospholipids in M-VLDL	21.5 ± 1.3	%	300	-0.38 [-0.48; -0.26]	<0.0001	300	-0.40 [-0.52; -0.27]	<0.0001	297
%Cholesterol in M-VLDL	30.9 ± 5.9	%	300	-0.42 [-0.52; -0.31]	<0.0001	300	-0.44 [-0.55; -0.31]	<0.0001	297
%Triglycerides in M-VLDL	47.6 ± 7.1	%	300	0.41 [0.31; 0.52]	<0.0001	300	0.43 [0.30; 0.55]	<0.0001	297
%Phospholipids in S-VLDL	21.6 ± 0.9	%	300	-0.39 [-0.50; -0.28]	<0.0001	300	-0.42 [-0.54; -0.29]	<0.0001	297
%Cholesterol in S-VLDL	42.9 ± 3.1	%	300	-0.33 [-0.44; -0.21]	<0.0001	300	-0.34 [-0.47; -0.20]	<0.0001	297
%Triglycerides in S-VLDL	35.5 ± 3.9	%	300	0.34 [0.23; 0.45]	<0.0001	300	0.36 [0.23; 0.49]	<0.0001	297
%Phospholipids in XS-VLDL	29.4 ± 0.7	%	300	0.33 [0.23; 0.44]	<0.0001	300	0.36 [0.25; 0.48]	<0.0001	297
%Cholesterol in XS-VLDL	51.8 ± 2.5	%	300	-0.40 [-0.50; -0.30]	<0.0001	300	-0.43 [-0.54; -0.31]	<0.0001	297
%Triglycerides in XS-VLDL	18.7 ± 1.8	%	300	0.42 [0.32; 0.52]	<0.0001	300	0.44 [0.33; 0.56]	<0.0001	297
%Phospholipids in IDL	23.6 ± 0.5	%	300	0.099 [-0.023; 0.23]	0.087	300	0.11 [-0.024; 0.26]	0.077	297
%Cholesterol in IDL	65.0 ± 2.0	%	300	-0.39 [-0.49; -0.29]	<0.0001	300	-0.43 [-0.55; -0.31]	<0.0001	297
%Triglycerides in IDL	11.4 ± 1.8	%	300	0.42 [0.32; 0.52]	<0.0001	300	0.46 [0.34; 0.57]	<0.0001	297
%Phospholipids in L-LDL	20.3 ± 0.6	%	300	-0.19 [-0.31; -0.059]	0.0011	300	-0.20 [-0.34; -0.061]	0.0012	297
%Cholesterol in L-LDL	70.3 ± 1.3	%	300	-0.30 [-0.41; -0.20]	<0.0001	300	-0.34 [-0.46; -0.22]	<0.0001	297
%Triglycerides in L-LDL	9.39 ± 1.51	%	300	0.34 [0.25; 0.44]	<0.0001	300	0.38 [0.26; 0.49]	<0.0001	297
%Phospholipids in M-LDL	23.3 ± 0.6	%	300	-0.24 [-0.35; -0.11]	<0.0001	300	-0.25 [-0.38; -0.11]	<0.0001	297
%Cholesterol in M-LDL	69.1 ± 1.3	%	300	-0.18 [-0.29; -0.072]	0.0020	300	-0.20 [-0.34; -0.081]	0.0016	297

%Triglycerides in M-LDL	7.66 ± 1.29	%	300	0.28 [0.18; 0.38]	<0.0001	300	0.31 [0.19; 0.42]	<0.0001	297
%Phospholipids in S-LDL	29.9 ± 1.0	%	300	-0.064 [-0.17; 0.060]	0.27	300	-0.047 [-0.17; 0.084]	0.46	297
%Cholesterol in S-LDL	63.9 ± 1.6	%	300	-0.23 [-0.34; -0.12]	<0.0001	300	-0.26 [-0.38; -0.14]	<0.0001	297
%Triglycerides in S-LDL	6.14 ± 1.01	%	300	0.42 [0.32; 0.52]	<0.0001	300	0.45 [0.33; 0.57]	<0.0001	297
%Phospholipids in XL-HDL	50.6 ± 2.0	%	300	-0.12 [-0.23; -0.006]	0.046	300	-0.10 [-0.22; 0.023]	0.11	297
%Cholesterol in XL-HDL	44.8 ± 1.5	%	300	-0.14 [-0.26; -0.023]	0.016	300	-0.17 [-0.31; -0.039]	0.0074	297
%Triglycerides in XL-HDL	4.68 ± 1.42	%	300	0.31 [0.21; 0.43]	<0.0001	300	0.32 [0.20; 0.47]	<0.0001	297
%Phospholipids in L-HDL	47.4 ± 1.6	%	300	0.28 [0.16; 0.41]	<0.0001	300	0.30 [0.16; 0.45]	<0.0001	297
%Cholesterol in L-HDL	45.7 ± 3.3	%	300	-0.35 [-0.45; -0.26]	<0.0001	300	-0.37 [-0.49; -0.26]	<0.0001	297
%Triglycerides in L-HDL	6.87 ± 2.80	%	300	0.24 [0.16; 0.34]	<0.0001	300	0.26 [0.17; 0.38]	<0.0001	297
%Phospholipids in M-HDL	46.9 ± 1.2	%	300	0.40 [0.28; 0.51]	<0.0001	300	0.43 [0.29; 0.56]	<0.0001	297
%Cholesterol in M-HDL	43.5 ± 3.7	%	300	-0.30 [-0.42; -0.20]	<0.0001	300	-0.34 [-0.46; -0.22]	<0.0001	297
%Triglycerides in M-HDL	9.52 ± 2.98	%	300	0.22 [0.11; 0.33]	0.00014	300	0.25 [0.12; 0.38]	<0.0001	297
%Phospholipids in S-HDL	56.5 ± 1.8	%	300	-0.015 [-0.13; 0.10]	0.80	300	-0.022 [-0.15; 0.11]	0.73	297
%Cholesterol in S-HDL	35.5 ± 2.2	%	300	-0.16 [-0.27; -0.050]	0.0051	300	-0.18 [-0.31; -0.051]	0.0044	297
%Triglycerides in S-HDL	7.98 ± 1.87	%	300	0.20 [0.085; 0.31]	0.00045	300	0.23 [0.10; 0.36]	0.00019	297
